# Barriers and facilitators to establishing Early Psychosis Intervention (EPI) services in remote and rural communities across Canada

**DOI:** 10.1371/journal.pone.0340888

**Published:** 2026-01-13

**Authors:** Bushra Khalid, Ashley Forbes, Kaya Radjah, Skye Barbic, Kamyar Keramatian

**Affiliations:** 1 Faculty of Medicine, University of British Columbia, Vancouver, BC, Canada; 2 Vancouver Coastal Health Authority, Vancouver, BC, Canada; 3 Department of Occupational Science and Occupational Therapy, University of British Columbia, Vancouver, BC, Canada,; 4 Department of Psychiatry, University of British Columbia, Vancouver, BC, Canada; University of Missouri School of Medicine, UNITED STATES OF AMERICA

## Abstract

**Aims:**

There is a paucity of data on how Early Psychosis Intervention (EPI) services adapt to the challenges of rural practice, including geographical isolation and provider shortages. This qualitative study seeks to fill this gap by exploring the experiences of EPI programs in rural and remote communities in Canada.

**Methods:**

An introductory email was sent to 117 EPI contacts, inviting them to first complete a survey and then participate in a semi-structured interview if their program contained a rural component. The interviews were conducted between June 2023-January 2024 via Zoom or phone by a member of our research team, while another member took detailed notes. Qualitative data was analyzed using thematic analysis.

**Results:**

Twenty-five representatives from seventeen distinct EPI programs with a rural and remote component across seven Canadian provinces and one Canadian territory participated in the interviews. Several barriers to establishing EPI programs in rural and remote areas were identified, including limited access to care, challenges in referral processes, geographical and technological constraints, and funding limitations. Facilitators of successful program delivery included the use of virtual care, standardized care models, strong leadership and teamwork, and adaptive resourcefulness in addressing local needs.

**Conclusion:**

Identifying and addressing these barriers and leveraging facilitators can enhance the accessibility and effectiveness of EPI service delivery in rural and remote areas.

## Introduction

Psychosis is a clinical condition marked by a significant disruption in reality-testing, often manifesting as hallucinations, delusions, and disorganized thinking, which impair a person’s ability to function [1]. It commonly arises in the context of psychiatric disorders such as schizophrenia, bipolar disorder, and substance-induced psychotic disorder [2,3]. Psychotic symptoms are linked to a reduced quality of life, shortened life expectancy, and an increased risk of both mental and physical health issues [4–6]. The onset of psychosis typically occurs in late adolescence or early adulthood, a critical period when individuals face important life transitions, such as completing education, forming relationships, and entering the workforce [7]. Early identification and intervention during this stage are vital, as they can significantly improve long-term recovery prospects and enhance daily functioning and quality of life [8].

Early Psychosis Intervention (EPI) programs are community-based, multidisciplinary clinical services designed to provide early identification, thorough assessment, and individualized treatment for individuals experiencing the initial stages of psychotic disorders. The first program of this kind, the Early Psychosis Prevention and Intervention Centre (EPPIC), was established in Melbourne in early 1990s [9] followed by the creation of similar programs in other parts of the world, including Canada. Research from these programs, which are exclusively located in well-resourced urban areas, has consistently shown their effectiveness in improving outcomes such as treatment adherence, hospitalization rates, relapse prevention, symptom severity, quality of life, and overall functioning, when compared to traditional treatment approaches [10,11]. However, there is a paucity of data on how EPI services are provided in rural and remote areas, and how programs in those areas adapt to the unique demands of rural practice such as geographical isolation and shortage of specialized mental health care providers. For instance, research in northern British Columbia has identified barriers such as the need for clinician schedule flexibility, difficulties in transitioning clients to appropriate programs, and the impact of mental health stigma in rural communities [12]. Another study highlighted resource-related constraints in rural northern Ontario, including smaller budgets, fewer full-time equivalent clinicians, and limited client volumes, which hinder adherence to provincial EPI guidelines [13]. While these studies provide important localized insights, a broader, nationwide understanding of rural EPI service delivery remains lacking.

In rural areas (defined by Statistics Canada as communities outside the commuting zone of urban centres with populations greater than 10,000) [14], individuals with early signs of psychosis may face unique challenges in accessing timely care, and the effectiveness of EPI programs in these settings may differ due to contextual factors such as transportation barriers, small or dispersed populations, and logistical difficulties in coordinating care between various disciplines [15]. These challenges necessitate a deeper understanding of how EPI programs adapt to the specific needs of rural populations and the barriers they face in delivering care. Moreover, rural healthcare providers may have limited experience or training in the etiology and unique presentations of psychosis, which could further complicate the early identification and treatment of these conditions [16]. Due to these challenges, it is crucial to examine how EPI programs in rural Canada are functioning by studying both facilitators and barriers to effective service delivery. This knowledge is important for ensuring that individuals in rural and remote areas receive the same high-quality care that is available in urban settings.

This qualitative study seeks to fill a gap in the literature by exploring the experiences of Early Psychosis Intervention (EPI) programs in rural communities in Canada. Using online surveys and semi-structured interviews, the study will examine how these programs are structured, how interdisciplinary teams function, and also identify facilitators and barriers to care delivery within these rural settings.

## Methods

Ethics approval for this study was obtained from the University of British Columbia Behavioural Research Ethics Board (H23-01091).

### EPI program representatives

This study was conducted using a mixed-methods sequential explanatory design, a recognized approach in which qualitative inquiry is used to clarify and deepen understanding of quantitative findings. This design was well suited to our objective of exploring barriers and facilitators in rural and remote EPI programs, despite the added time and analytic complexity involved [17]. We first implemented a national survey to gather broad descriptive data on the delivery of EPI services in rural and remote settings across Canada. Findings from the survey were then used to inform the interview phase, including the development of the interview guide and the selection of key topics for deeper exploration during interviews with program representatives. For example, the Qualtrics survey helped us to understand the general make up and structure of the team (i.e., psychiatry time, discipline make up, general workflows), which prompted us to utilize the interview to ask about the comprehensiveness of services, the successes and challenges with their model, and what teams would do differently if they had the ability. The qualitative data offered a contextualized, in-depth understanding of participants’ experiences, thereby helping to interpret and enrich the survey results.

Participant recruitment began on 21/06/2023 and ended on 14/11/2023. To identify EPI programs across Canada, we conducted a comprehensive web-based search of EPI services in all Canadian provinces and territories. This included reviewing publicly available directories, provincial health authority websites, and relevant organizational listings. Within the Qualtrics survey and interviews, participants were asked to direct the research team to other relevant programs and/or contacts that may be providing EPI services in rural and remote communities across Canada. Given the absence of a universally accepted definition of “rural and remote” in the context of Canadian mental health services, we adopted a pragmatic, participatory approach: during outreach, we asked representatives from each EPI program whether their team served rural and/or remote populations. Programs were included in our study if they self-identified as having a rural or remote component to their service delivery. This self-identification approach reflects the diversity of rural and remote contexts across provinces and territories. To help ensure broader coverage, we also asked participants to forward information about any other EPI programs they were aware of that serve rural or remote populations.

We used purposive sampling, inviting program representatives with direct experience in delivering EPI services in rural and remote settings. Participation was limited to representatives who were both available and willing to take part during the recruitment period. While we did not formally assess saturation, our team met weekly to code the data and understand recurring patterns across interviews suggested that the themes were reasonably stable. An introductory email, which explained ongoing efforts by the Coastal EPI Program – a regional specialized service for early psychosis within Vancouver Coastal Health – to engage rural and remote mental health and substance use programs in the process of developing collaborative care models for EPI clients within their communities, was sent to 117 Canadian EPI contacts. The email outlined the following requests to potential participants: (1) to participate in our voluntary EPI Qualtrics Survey, (2) to indicate their interest in participating in a semi-structured interview, and (3) to forward any information about other known EPI rural and remote services within Canada to our team. Clinicians who were unable to complete the survey due to staff changes or scheduling challenges were asked to forward the introductory email and Qualtrics link to fellow clinicians or colleagues they believed would be interested in participating in the study. Informed consent was obtained through the online Qualtrics survey. Participants were provided with an information sheet at the beginning of the survey outlining the purpose of the study, data confidentiality, and the voluntary nature of participation. Consent to participate in the survey was implied through its completion. Participants who expressed interest in a follow-up interview indicated their consent to be contacted by responding “yes” to the question, “Would you be willing to have a brief 30-minute phone/Zoom call with our team regarding rural and remote EPI services?”. Separate consent was verbally reconfirmed at the beginning of each interview.

### EPI qualtrics survey and interviews

The EPI Qualtrics Survey (see Table 1) was designed for currently existing EPI programs across Canada with the intent of collecting generalized information about team makeup, workflow, inclusion and exclusion criteria, referral statistics, geographic descriptions, and specific service types made available by each program. The survey contained a total of 23 questions. This survey was designed to take approximately 15 minutes for the recipient to complete. Semi-structured Zoom or telephone interviews using the EPI Rural and Remote Services Questionnaire (see Table 2) were conducted to collect data from Canadian EPI program team leads, clinical planners, clinicians, or managers, whose programs provide EPI services to rural and remote communities. The interviews were conducted between June 2023-January 2024 via Zoom or phone by a member of our research team (AF) who also serves as an EPI team lead. Another member of the research team (KR) took detailed notes during each interview. The interviews were not audio recorded, in line with the study’s approved protocol. This decision was informed by both methodological and practical considerations; namely, to promote participant openness when discussing service delivery challenges, and to accommodate resource limitations related to transcription. Instead, all data were captured through real-time notetaking using a password-protected laptop in accordance with institutional privacy policies. As an incentive, two participants received a CAD 50 gift card, awarded through a random draw.

**Table 1:** EPI Qualtrics Survey (Online).

Your name (used only to arrange optional semi-structured interviews (see question #22) and/or to be entered into our prize draw)
The full name of your program
Your position
How many Full Time Equivalents (FTEs) non-physician staff do you have in your team?
What is the staffing compliment of your team? (i.e., nursing, social work, OT, etc.)
How many Full Time Equivalents (FTEs) psychiatrists do you have in your team?
Do you consider your program a full-fidelity EPI program? Yes; No; Unsure
What are the geographical boundaries of your EPI program?
What is the total population of the area your EPI program serves?
What are the minimum and maximum age limits for the clients?
What are the other eligibility criteria (diagnoses, duration of illness etc.) for clients to access services?
What are the exclusion criteria (i.e., affective psychosis, substance use disorder, developmental disability, or any others)?
Approximately what percentage of referrals to your program come from inpatient settings?
Approximately what percentage of referrals to your program come from emergency departments?
Approximately what percentage of referrals to your program come from the community?
How many clients do you have in your team?
What is the average wait time for clients to be seen by a case manager?
What is the average wait time for clients to be assessed by a psychiatrist?
What services does your program provide? (Select all that apply): Psychiatric assessment; Psychiatric follow up; Case management; Family counselling; Family education; Substance use counselling; Cognitive remediation; Group psychoeducation for clients; Group psychoeducation for families; Metabolic monitoring; Other (please describe): ______
What would best describe the geographical area that your EPI program is meant to serve? Urban only; Urban and rural/semi-rural; Semi-rural/rural only
Does your EPI program currently provide services to rural, semi-rural, or remote communities? Yes, full service; Yes, limited service; No
If YES, would you be willing to have a brief 30-minute phone/Zoom call with our team regarding rural and remote EPI services? (Yes/No)
Can you name other Canadian EPI programs who currently offer rural services?

**Table 2:** Semi-structured interview probing questions.

Can you tell us about the EPI services that you offer to rural and remote communities?
How long have you been offering EPI services to rural and remote communities?
What was the process of establishing the program?
What are your eligibility criteria to access this service?
What is the geographic region and estimate population size of the region you serve?
What is your current service model for providing services to rural and remote communities?
6.a. Do you offer full EPI services?
6.b. Do you offer psychiatric consultation only, or do you offer follow-ups with the psychiatrist as well? Are visits (consultations and follow-ups) mainly virtual, over the phone, or in person?
6.c. Do you ever provide a physician-to-physician consult service? If yes, can you tell me more about it?
6.d. What about case management?
Approximately how many psychiatric consults a month do you provide?
What does your program’s workflow look like for referrals, consultations, recommendations, and communication?
What has gone well with this service? Have there been any challenges since starting the program?
If given the opportunity to set up this service again, what would you do differently? Do you have any advice for our new EPI Coastal team?
Have you had any opportunities to evaluate the service? Did you hear any feedback from patients and care providers?
Do you know of any other EPI programs that are providing services to rural/remote communities in your province or in Canada?

### Qualitative data analysis

Interview data were analyzed thematically using NVivo (version 14.0) following an inductive approach outlined by Braun and Clarke’s six step method [18]. We chose an inductive thematic analysis approach to allow themes to emerge directly from the data, rather than being shaped by pre-existing theoretical frameworks or assumptions. Given the limited research exploring service providers’ experiences in rural and remote communities, an inductive method was preferred to ensure that participants’ perspectives guided the analysis and that novel or unexpected insights could be captured authentically. The data were initially coded by the lead author (BK). Subsequently, all transcripts and codes were independently reviewed and then collaboratively refined by three other research team members (SB, AF, and KK) during weekly meetings to ensure consistency and rigor in the coding process. While formal intercoder reliability was not calculated, we used these weekly team meetings to resolve discrepancies and reach consensus on coding decisions. To support reflexivity, we maintained analytic memos and engaged in ongoing discussions to reflect on our assumptions, positionality, and potential biases throughout the analysis. In these meetings, the team iteratively reviewed the data and refined the themes to strengthen the credibility and validity of the findings. Collectively, the team has extensive clinical and applied experience with EPI health services research and qualitative research. We read the transcripts multiple times and initial memos taken. A final list of themes developed from the initial coding process was compiled and subsequently used for manuscript preparation.

## Results

### EPI program interviews and site characteristics

**Fig 1:**
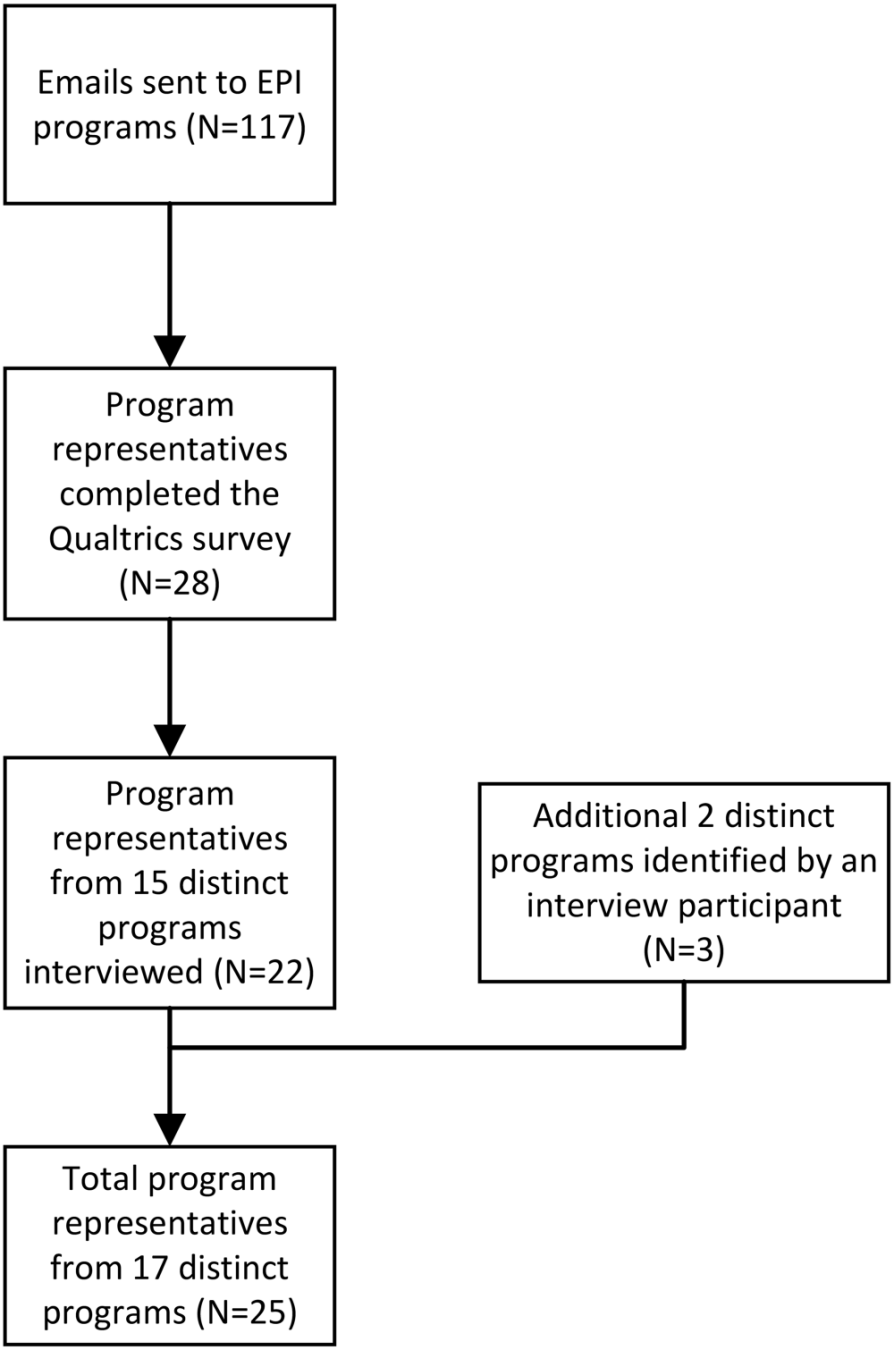
Flowchart depicting the recruitment process including the number of EPI program representatives who completed the Qualtrics survey and those who participated in the interview.

The recruitment flowchart is presented in Fig 1. The Qualtrics survey was completed by 28 responders who self identified as nurses/clinical nurse educators (n = 8), program managers (n = 7), clinical coordinators (n = 5), EPI clinician (n = 4), social workers (n = 1), community mental health worker (n = 1), evaluation analyst (n = 1), and scientist (n = 1). The interview sample consisted of 21 interviews with 25 representatives from EPI programs serving rural and remote communities across Canada. These 25 representatives came from 17 distinct programs. Three programs had more than one representative participate in the interviews: one program from BC had three representatives, another program from BC had two representatives, and one program from QC had two representatives. For these interviews, we used a dyadic/triadic interactive interviewing approach, allowing participants to respond to and build on each other’s perspectives [19,20]. These joint interviews followed the same semi-structured guide as individual interviews, and the responses were analyzed together.

Of the 25 interview participants, 20 had completed the Qualtrics survey prior to their interview. The interview sample included EPI programs across seven Canadian provinces and one Canadian territory: British Columbia (BC), Alberta (AB), Manitoba (MB), Ontario (ON), Nova Scotia (NS), Newfoundland and Labrador (NL), Quebec (QC), and the Yukon Territory (YT), while respondents to the Qualtrics survey represented five provinces and one territory (BC, AB, MB, ON, NS, YT). This discrepancy reflects three interview participants (two from QC and one from NL) who did not complete the survey (see Table 3 for details). Results from the interviews revealed several themes which were categorized as barriers or facilitators to developing EPI programs in remote and rural communities.

**Table 3:** Characteristics of Canadian Early Psychosis Intervention (EPI) programs with a rural and remote component based on the Qualtrics survey.

Province/ Territory	Interviewee and Position	Program Constituents	# FTE Psychiatrists	# FTE non-physician staff	Approximate Population Size of Region Served	# Psychiatric Consults Monthly	# of Clients	Avg wait time to be seen by case manager	Avg wait time to be seen by psychiatry
**British Columbia**	Clinical coordinator*	1. Psychiatrist 2. Nurse practitioner 3. Clinical coordinator 4. Social workers 5. Life skills worker 6. Nursing 7. Nurse Educator	1.0	11	290,000	2-3	~70	48 hours	NA
**British Columbia**	CNE/Mental Health and Substance Use Community Liaison	1. Nursing 2. Registered clinical counsellor 3. Social worker	0 Will refer to a one-time consultative virtual psychiatric service	2	6500	NA	NA	NA	2-3 weeks
**British Columbia**	Mental Health Clinician	1. Mental health clinician	Have some virtual access to EPI psychiatrist in closest urban community	1	60,000	5-8	10	2-3 business days	NA
**British Columbia**	Team Lead	1. Nursing 2. Social worker 3. Allied health 4. Primary care assistant	2	26	Unsure	NA	80	2-3 weeks	5 months
**British Columbia**	Mental Health Clinician	1. Nursing 2. Occupational therapist	0.1	0.6	70,000–100,000	NA	20	< 2 days	Within 2 weeks
**British Columbia**	Clinical Educator	1. Nursing 2. Social worker 3. Registered clinical counsellor 4. Occupational therapist 5. Youth care worker 6. Recreation therapist 7. Group and family therapist 8. Intake concurrent disorders therapist 9. Psychiatrist	Unsure	13	Unsure	NA	Unsure	Unsure	Unsure
**Alberta**	Referral Coordinator	1. Psychiatrists 2. Nursing 2. Social workers 3. Addiction counsellors 4. Occupational therapists 5. Psychologists 6. Psychometrist 7. Care manager 8. Clinical manager	1.2	Difficult to quantify (6 Mental health teams – varying FTE. 2 managers – shared amongst several programs), referral coordinator – 1FTE), admin – 0.5FTE), psychologist II – 1 FTE	1 million	Not many, approx. a few a month	175	Within 1 week	Approx 1 month
**Manitoba**	Community mental health worker/ Registered psychiatric nurse*	1. Nursing 2. Activity worker	1.0	2	170,899	3-4	50	1-3 days	1-3 weeks
**Ontario**	Manager	1. Psychiatrist 2. Nursing 3. Peer support workers 4. Case managers 5. Family workers	1.0	9	300,000	12-13	NA	2 weeks	Within 1 month
**Ontario**	Manager	1. Nursing 2. Community mental health counsellor	0.2	2	61,735	8-10	30	24-48 hours	3-4 weeks
**Ontario**	Team Lead	1. Nursing	0 Utilize psychiatrists connected to hospital – moving towards having psychiatrists attached specifically to the EPI program	3	105,000	3.5	50	72 hours	1-2 weeks
**Ontario**	Registered Nurse	1. Psychiatrist 2. Nursing	0.2	1	21,000	Approx once every two months (only see people on Thursday mornings)	17	1-2 months	Up to 1 year
**Ontario**	Director of Services	1. Nursing 2. Social worker 3. Occupational therapist 4. Peer support 5. Family support 6. Intake coordinator	1.0	15.6	1 million	Not a lot, approx. 10–20	NA	6-9 months	3 months
**Ontario**	Director of Services	1. Case mangers 2. occupational therapist 3. Clinical lead 4. Nursing 5. Peer specialist 6. Family worker	0.2	12 FTE (Clinical staff) + 1 FTE manager + 1 FTE admin	1.25 million	NA	85	3-6 months	3-6 months
**Ontario**	Registered Nurse	1. Nursing	0 No clarification provided	3	Unsure	NA	75	2-3 days	Usually already assessed
**Nova Scotia** **(Province representative)**	Provincial Coordinator	1. Psychiatrist 2. Nursing 3. Community outreach workers 4. Occupational therapist 5. Social Worker	NA	NA	1 million (Approx half of this is rural/remote)	85	unknown	1-2 weeks	Depends on area
**Yukon Territory**	Supervisor (Social Worker)	1. Nursing 2. Social worker 3. Outreach workers	1	10	45,148	2-4	17 EPI, 78 Psychiatric Outreach Program (these two teams are integrated)	No wait times	2 weeks

*Representative spoke on behalf of multiple EPI programs.

EPI, Early Psychosis Intervention; FTE: Full Time Equivalent.

### Barriers

Several barriers to successfully setting up EPI programs in these contexts were identified.

#### Theme 1: Lack of access to care.

One of the most prominent themes that was elicited from across the interviews was limited access to care in the form of shortage of psychiatrists, minimal outpatient services, lengthy waitlists, and lack of allied healthcare. In rural communities, participants reported that patients are often initially assessed and referred to psychiatry by their primary healthcare providers. However, one program representative emphasized that their program “*didn’t have enough psychiatry time for everyone to be seen and therefore had to push back from community referrals*.” Participants noted that this resulted in lengthier wait times for individuals who did have their referral approved, which was *“a huge challenge for the program and was difficult to navigate and ensure this didn’t happen.”* Programs highlighted that these longer wait times are a significant barrier to recovery given the importance of early treatment in their disease. Another program representative similarly reported that *“the psychiatrist in the team does 15-minute check-ins, but otherwise is there for consults and medications; patients don’t see them every month.”*

Once patients have been stabilized within the EPI programs, participants noted that they often require supplemental treatment or support from outpatient programs, whether that is addictions support, group therapy, or housing resources. However, many provinces have found it to be a real challenge to *“find the RIGHT services for folks in resources…for example there is no foundry yet despite funding being approved.”* Participants noted a downstream effect of not having facilities to support clients who are not appropriate for EPI resources or to ensure the initial stabilization by EPI programs is solidified. One program representative revealed “*it has been a challenge creating community around client; for example, there is not a nurse dedicated to bloodwork/metabolic monitoring/injection.”* As such, participants across all provinces and one territory shared that there has been significant frustration from service providers over how to best support their clients over time with the limited resources available.

#### Theme 2: Referral process and program mandate alignment.

Although the majority of programs clearly outlined exclusion and inclusion criteria, our data suggest that ensuring that appropriate referrals are received and maintaining a clear referral process has remained a challenge for many programs in rural areas. One clinical coordinator highlighted that *“there are variable ways that clinics interpret eligibility,”* which often results in a discrepancy between which programs will accept a certain type of patient. Another program representative found that due to a high number of referrals that misaligned with the program mandate, they had to *“be clear with what the service can and cannot provide.”*

One program representative noted that *“a very small percentage of referrals were appropriate,”* while another program manager stated that their team *“promoted people sending referrals but got inappropriate referrals”* with multiple clients presenting with personality concerns. All participants noted that this has significant impacts on how quickly an individual can receive treatment, as programs are forced to spend administrative resources on assessing long waitlists.

#### Theme 3: Geographical and technological barriers.

Many program representatives highlighted the difficult aspects of providing adequate care to EPI patients residing in remote locations, including technology access, transportation issues, limited crisis intervention, and minimal group therapy options. While virtual care has been a valuable tool for assessing patients remotely, participants reported that this method falls short when clients do not have the necessary resources to use it. One program representative mentioned that despite advancements in technology, *“it can be very difficult to contact clients who are far away.”* Participants noted that this often leads to lapses in continuity in care, with *“patients commonly getting lost to follow-up.”* Participants reported that distance is the most significant roadblock to treatment in a condition which demands close monitoring and possible medication adjustments. Another program representative reflected on the consequences of this, which is that *“being remote can cause a safety issue, and we really need someone located close by.”*

Participants also highlighted the importance of regular group therapy in treating psychosis but acknowledged the difficulties in facilitating patients to attend. Despite many programs implementing consistent outreach work, participants noted that the significant geographical distance between communities and treatment providers remains a hurdle to overcome when providing effective care.

Participants also noted that the issue of overcoming geographical distances between patient populations is compounded by varying degrees of access to technology, which may otherwise help deal with these issues. A program representative states: *Many people don’t have landlines and if they have cellphones, they want to use apps. However, service providers can’t talk through these apps and are therefore limited to text.”* Moreover, programs noted that even in cases where clients may have cellphones and landlines, they are still limited based on access to internet: *“A challenge has been having connectivity to internet while providing virtual services.”*

#### Theme 4: Finances and funding.

A fourth theme suggested by study participants was lack of financial resources and funding which led to difficulties in providing adequate services. Participants highlighted that a key component of providing competent care is having well-trained staff who have undergone the appropriate training to manage and treat patients with psychosis. One program representative stated that although they were doing their best to offer mental health training to new staff, *“training is limited based on funding.”* Furthermore, even when trained staff are part of the team, lack of available funding makes it difficult to hire enough mental health workers to cover the needs of increasing number of patients: “*Great to be able to provide services to clients as needed, but not always feasible with funding and demands of caseloads.”* Another program representative echoed similar thoughts, stating that programs are often *“told they cannot have a casual worker, so they don’t have back-up.”* Participants across this study emphasized that this has implications for worker burnout, sustaining workforce in rural and remote communities, and potentially declining quality of patient care.

### Facilitators

Analysis of the interviews revealed many themes indicative of factors that have aided EPI programs in successfully delivering adequate care to patients dealing with early psychosis.

#### Theme 1: Virtual care.

Given that rural and remote communities are often very far from the main hub communities of EPI programs, many interviewees highlighted the value of using virtual care to provide care. As stated by a program representative, *“with distances involved it has helped to do it virtually, and although we do have a hybrid model, it is possible to do clinics virtually”*

with another program using a model where *“consults are performed in person and then follow-up care can be virtual.”* While patients may have to travel for their initial assessment, technology has facilitated the convenience of patients not having to do repeated travel, which can increase patient engagement with their care team. Moreover, some participants noted that “*the clinicians have adapted to virtual care well and are able to connect with clients without making it too onerous.”* As one program representative noted, this modality is especially useful with psychiatry time, given the limited number of specialists available for each region.

#### Theme 2: Standardized model of care.

One theme consistently highlighted throughout the interviews was the progressively standardized model of care that programs are transitioning to using. Programs revealed that in the past, services were fragmented and there were no consistent guidelines for management of psychosis. However, one program representative revealed that their recent transition to a standardized care model has increased patient care and family satisfaction: “*Moving to a standardized model of care has been massively beneficial. The families provided positive feedback and noted that the client is getting what other clients are getting, and this care is based in research.”* Another program representative noted similar positive results when discussing their team’s use of the standardized NAVIGATE approach to care, saying that *“the families are really happy about the NAVIGATE approach, likely because they are very included in the care.”* A third program representative discussed the different ways they have been able to positively instill change within their care protocols, stating that they “*developed a manual with individual resiliency training, which was written for people who have never worked in EPI which contains clinical guidelines.”*

#### Theme 3: Supportive leadership and team/relational approach.

A significant facilitator to establishing EPI programs in rural/remote communities has been ensuring programs have leadership that represents the client population and oversees the daily workings of the program. Specifically, one program representative stated that their team members “*appreciate having a coordinator who is someone in lead role overseeing and bringing them together by getting insights and having annual feedback conferences.”* Furthermore, programs highlighted the importance of having leadership which represents the clients in each region to best serve their individual interests: “*Finding* r*epresentation and leadership in each zone and finding folks with interest has been important, since they know geography of land… they know it the best which makes advocacy easier because there is tight knit community.”* Representatives also specifically focus on the importance of connecting with Indigenous communities to optimize engagement with their programs: “*Indigenous communities have a hard time engaging with healthcare system, so visiting communities can be helpful for connecting a face and then following up with Zoom.”* This was further emphasized by another program who realized the importance of collecting insight from remote communities to help plan an approach which would be most beneficial. They stated that *“making the effort to go out and connect with the communities has been very helpful. The program realizing the reality of their circumstances has helped us to plan a service that’s going to be helpful.”*

#### Theme 4: Adaptive resourcefulness/education.

Several program representatives revealed resourceful tactics for optimizing their services. For example, to overcome geographical barriers, “*clinicians cluster their care depending on towns most client meetings are in, especially when towns are a couple hours apart.”* Several program representatives also placed a heavy emphasis on their work in improving education for clinicians to streamline the referrals process: “*Education is being provided to rural clinicians to understand who is appropriate for program.”* Improving education for clinicians has also been invaluable in expanding their knowledge around the precise issues their patient population experiences, such as substance use: *The team has done a lot of traveling and education workshops which has been helpful for them learning the extent of the challenges and the extent of the substance use issues.”*

## Discussion

To our knowledge, this is the first study to explicitly examine Canada-wide barriers and facilitators faced by Early Psychosis Intervention (EPI) programs in rural and remote communities across the country. Previous research has mostly focused on barriers and strengths within individual provinces, such as northern British Columbia, where key challenges identified included the need for clinician schedule flexibility, smooth transitions to appropriate programs, and societal stigma surrounding mental health [12]. Similarly, Durbin et al. (2016) explored factors hindering rural EPI programs in northern Ontario from following provincial guidelines, finding that lower budgets, fewer full-time equivalent clinicians, and fewer clients were significant barriers [13]. These earlier studies have largely concentrated on smaller regions, creating a gap in the literature regarding a nationwide, qualitative assessment of EPI programs and their challenges.

Our study stands out for its comprehensive, nationwide assessment of barriers and facilitators faced by EPI programs in rural and remote communities across Canada. We identified several key barriers to establishing EPI services, including limited access to care, financial constraints, and workforce shortages. These findings align with previous research, such as the study in rural Ontario, which highlighted that EPI services could better meet provincial standards if they had improved access to psychiatry and physical health monitoring [13]. Our results also reveal facilitators for providing EPI care in remote areas, such as the use of telepsychiatry for consultations and follow-ups—an approach also identified by Stain et al. (2008) as a viable method for early psychosis assessments and referrals [21].

Moreover, our study underscores the importance of transitioning from fragmented treatment models to standardized care models, like NAVIGATE, to ensure coordinated care across multidisciplinary teams. The NAVIGATE program, which focuses on comprehensive care for individuals experiencing their first episode of psychosis, emphasizes a team-based approach with a particular focus on involving the patient’s family in the recovery process [22]. The value of standardized treatment models has also been noted by Bedard et al. (2016), who found that using such models helped regulate both services and documentation across various EPI programs, ultimately improving quality assurance and program evaluation [23]. Beyond reinforcing existing literature, our findings also highlight novel facilitators to overcoming challenges in EPI care within the rural context, such as implementing supportive leadership to address the unique needs of each rural community and incorporating adaptative resourcefulness to group treatments in remote locations.

The interaction of the aforementioned factors offers valuable insights into avenues for improving service delivery and patient engagement in rural and remote Canadian locations.

### Standardized models of care: improving consistency and patient satisfaction

Several program managers emphasized the importance of transitioning from fragmented treatment approaches to standardized care models to ensure consistency in the services each patient receives. Many leaders specifically pointed to the effectiveness of hub-and-spoke models, which fostered coordinated care by strengthening connections between mental health workers, primary care providers, and community resources. This finding aligns with previous research showing that hub-and-spoke models help reduce relapse rates in first episodes of psychosis [24,25].

Beyond coordination, our study revealed that EPI programs that shifted from disjointed to standardized approaches, such as NAVIGATE, saw significant improvements in clinical outcomes and patient satisfaction. This was particularly evident in programs that involved families in every step of the treatment process. The NAVIGATE model integrates medication management, psychosocial interventions, and family dynamics to enhance both short- and long-term outcomes for individuals with early psychosis [22]. Program representatives in our study highlighted the positive feedback from patients and families regarding NAVIGATE, especially its emphasis on family involvement throughout the treatment. Programs found that this approach strengthened the therapeutic relationship and encouraged patients to actively engage in their care, leading to better adherence to treatment plans.

While standardization in early psychosis intervention (EPI) programs ensures that patients receive consistent care, it is crucial that the implementation of these standardized models is carefully tailored to the specific cultural and environmental contexts of the young people being served. Specifically, programs in our study found that for young individuals experiencing early psychosis, care cannot simply be standardized in terms of clinical protocols and treatment plans; it must also resonate with their lived experiences, cultural backgrounds, and social environments. This ultimately increases patient engagement with treatment plans.

Ultimately, the results from this study underscore the importance of adopting standardized care models in EPI services, not only to ensure consistency across treatment plans but also to provide culturally relevant care while engaging families as integral members of the multidisciplinary care team, ultimately optimizing patient outcomes.

### Geographical and technological barriers: a gradually improving issue

Many program leaders in our study emphasized the significant challenges EPI programs face due to limited availability of specialized services, long waitlists, and the travel burdens associated with geographical isolation. Geographic constraints often led to fragmented care, as patients were unable to attend appointments due to transportation issues, which in turn resulted in poor continuity of care or, in some cases, patients being lost to follow-up. Technological barriers, such as poor internet connectivity and lack of portable devices, further compounded these issues by making virtual consultations difficult for both patients and healthcare providers.

However, we found that a gradual focus on expanding technological infrastructure was a key solution to these challenges. Many programs implemented hybrid models that combined in-person assessments with virtual follow-ups. Several program managers reported that expanding the use of telepsychiatry led to significant improvements in patient engagement and continuity of care. This aligns with findings from Cheng et al. (2013), who concluded that videoconferencing is an effective way to increase the capacity for EPI services in rural areas [24]. Additionally, the reliability of telepsychiatry in psychosis assessments has been well-established, supporting its role in overcoming geographic barriers [21,26]. These findings underscore the potential of virtual care to increase access to services and reduce travel burdens for patients in remote communities.

It is worth noting that although technology has the potential to greatly enhance healthcare delivery in rural areas by reducing travel burdens and improving access, its effectiveness is limited for patients who lack the necessary devices, have poor internet connectivity, or face challenges with technological literacy, as described in our study and others [27–29]. While these challenges are not unique to rural settings, several barriers appear more pronounced in rural and remote communities where infrastructure is limited, and healthcare options are scarce. These factors emphasize the need for adaptable, culturally sensitive care models that account for both the geographic and demographic diversity of rural populations when integrating technological solutions. The dual role of technology as both a facilitator and a barrier highlights the need for adaptable, culturally sensitive care models that account for the geographic and demographic diversity of rural populations when integrating technological solutions.

### Workforce shortages and the role of community leadership

Program leaders identified staffing shortages, particularly among psychiatrists, nurses, and social workers, as a persistent barrier across many EPI programs. These shortages often led to long waitlists, with some patients ultimately falling through the cracks. The root cause of these staffing issues was insufficient funding, as many programs lacked the financial resources to expand their workforce. This aligns with findings from Durbin et al. (2016), which highlighted that limited funding in rural EPI services led to lower rates of implementation of provincial psychiatric service standards [13]. However, our study also revealed a unique insight: effective community leadership played a crucial role in overcoming these challenges. Programs with engaged leaders who understood the specific needs of their communities and fostered a collaborative team environment were better equipped to manage workforce shortages. Additionally, leaders who actively engaged with local communities, especially Indigenous populations, were more successful in building trust and improving service uptake. The literature supports this approach, noting that when efforts are made to understand unique barriers to treatment in the Indigenous context—such as stigma and mistrust of the healthcare system—youth are more likely to seek care [30]. This is especially relevant given that a known, primary barrier for implementing EPI programs within Indigenous communities is a lack of cultural safety within the service and treatment plans [31]. By enhancing clinicians’ knowledge of early psychosis, cultural competence, and unique needs of diverse communities, programs can better equip local clinicians to manage the complex needs of patients and implement best (evidence-base and wise) practices.

### Adaptive resourcefulness and the importance of education

Given the resource constraints faced by many programs, adaptive resourcefulness emerged as a novel, key facilitator in ensuring that EPI services remained accessible and effective. Programs developed innovative strategies to address logistical challenges, such as clustering care to reduce travel burdens for both clinicians and patients. As program leaders emphasized, these solutions were crucial for ensuring that patients could access care without facing excessive travel time or costs, particularly in rural and remote areas.

Furthermore, the data from this study highlighted the importance of training mental health workers to better screen for early psychosis. By improving screening accuracy, programs could streamline the referral process and reduce the time clinicians spent on inaccurate referrals. This finding aligns with Cheng et al. (2013), who noted that education programs for mental health workers help strengthen EPI knowledge and increase appropriate referrals [32]. Additionally, program representatives stressed the value of expanding education on specific risk factors for early psychosis, such as substance use, and how to address these issues with clients. By enhancing clinicians’ understanding of the factors contributing to early psychosis, they will be better equipped to target these issues and improve the overall effectiveness of EPI services.

### Implications

The findings from this study allow for several key implications to be extracted. Firstly, we discussed the importance of coordinated care between multidisciplinary health care providers. Our results show that the transition to standardized models of care has been instrumental in this regard by increasing communication between care teams while facilitating patient engagement and family satisfaction. Thus, policymakers and decision-makers should continue to encourage implementing standardized care to improve clinical outcomes and experiences.

Also, our data overwhelmingly encourage the use of telepsychiatry in early psychosis assessments and follow-ups, allowing patients and clinicians to overcome vast geographical isolation faced by many rural and remote communities. We urge program developers to invest in ongoing advancements in virtual care innovation to allow patients to access the often-limited number of mental health professionals in their region.

Finally, our findings also support the utility of strong leadership through comprehensive education in assessing early psychosis, which we found to be particularly important in fostering culturally competent care. Program managers should ensure their workforce is provided adequate education in the unique health challenges that rural and Indigenous communities face to connect with their clients and increase patient engagement with treatment plans.

## Limitations and future directions

There are several important limitations to consider when interpreting the findings of this study. First, the sample did not include all EPI programs across Canada, which means the results may not fully represent the diverse experiences of EPI teams nationwide. The total number of EPI programs with rural and remote services across Canada is not known; therefore, we were not able to accurately estimate what proportion of all eligible rural and remote EPI programs our study sample represents. A further consideration is that although introductory emails were sent to 117 Canadian EPI contacts, only 28 individuals completed the survey. This response rate reflects the fact that the survey and interview invitation specifically targeted programs that provide services to rural or remote communities—a subset of the broader national EPI network. Many programs contacted did not have a rural or remote service component and were therefore not eligible or did not perceive the study to be relevant to their context. Another limitation of our study is the reliance on program self-identification to define rural and remote service delivery. This approach may have led to differences in how programs understood and defined “rural” and “remote,” meaning that the degree of rurality or remoteness likely varied across programs. However, we felt this was a necessary and practical approach, given the diverse contexts across Canada, and it allowed us to include programs that might have otherwise been missed using a strict definition. Additionally, the interviews were not audio-recorded; instead, detailed notes were taken during each interview. This decision was made due to resource limitations related to transcription, as well as to promote openness among participants when discussing potentially sensitive challenges in delivering services in rural and remote settings. This approach may have led to the omission of certain nuances in the responses, as note-takers might have excluded “filler” words or assumed certain details were common knowledge. It also limited our ability to capture verbatim quotations and potentially heightened reliance on the interviewer’s subjective understanding of responses. However, as noted by Rutakumwa (2020), not using audio recordings can help ensure that interviewees feel more comfortable and provide more spontaneous, open responses [33]. This is supported by McMullin (2023), who highlighted that that while voice recordings can improve data accuracy and reduce interviewer bias, they can sometimes make participants feel uncomfortable and lead to self-censorship [34].

For future research, it would be beneficial to expand the sample to include a wider range of EPI programs across Canada, providing a more comprehensive and representative analysis. Incorporating audio recordings in interviews could also enhance the accuracy and richness of the data collected.

## Conclusion

Our study looks at the challenges and solutions for delivering early psychosis care across Canada. While programs face challenges such as workforce shortages, limited access to care, and geographic isolation, key factors including virtual care, standardized care models that allow for consideration of community context, supportive leadership, and flexibility have helped programs overcome these obstacles. By building on these strengths and addressing the challenges, EPI programs can improve their ability to provide timely, high-quality care, especially in rural and remote areas.

## Supporting information

S1 Appendix Consent information form. (PDF)

S2 Appendix COREQ (COnsolidated criteria for REporting Qualitative research) Checklist. (PDF)
