# Supplementary material for: Barriers and facilitators to establishing Early Psychosis Intervention (EPI) services in remote and rural communities across Canada
Source: PLoS One. 2026 Jan 13;21(1):e0340888. doi: 10.1371/journal.pone.0340888 (PMC12799007; doi:10.1371/journal.pone.0340888)
Supplement: S1 Appendix — (PDF) [file pone.0340888.s001.pdf]

Main Study Consent Form
EPI Coastal Rural & Remote Community Project

I. Study Team

Principal Investigator:
- Dr. Kamyar Keramatian - Psychiatrist | Clinical Assistant Professor | UBC Psychiatry

Co-Investigators:
- Ashley Forbes - EPI Coastal Team Lead | ashley.forbes@vch.ca | (604) 219-6803
- Kaya Radjah - EPI Coastal Practicum Student | s51654@vch.ca

II. Sponsor

This research received no specific funding. Our study team has no relevant financial interests to disclose.

III. Invitation and Study Purpose

You are being invited to take part in a brief Qualtrics survey and potentially an informational interview should your program currently be providing EPI services in rural communities. Our team wants to: (1) understand the status of EPI services in Canada, as well as more specifically rural and remote EPI services, (2) understand the facilitators and barriers of care for individuals with early psychosis who live in rural and remote communities, and (3) apply knowledge gathered from data collection to EPI Coastal practices.

We aim to assist individuals in the rural and remote communities referred to EPI Coastal:

1. Access appropriate and adequate treatment recommendations.
2. Increase service providers' knowledge and confidence in treating individuals in early psychosis.
3. Provide appropriate support and education to clients and families regarding psychosis.

This should support clients in working towards goals such as:

1. Regain the ability to think clearly and feel like their usual self, succeed at school or work and enjoy satisfying relationships.
2. Re-engage with goals, ambitions, and developmental milestones.
3. Reduce social isolation, depression, and the potential risk for harm to self or others.

IV. Study Procedures

The online Qualtrics Survey has been designed for currently existing EPI programs across Canada with the intent of collecting generalized information about team makeup, workflow, inclusion and exclusion criteria, referral statistics, geographic descriptions, and specific service types made available by each program. This survey should take approximately 10-15 minutes for the recipient to complete.

If your team is providing EPI services to rural and remote communities, you will be invited to participate in a 30-minute semi-structured interview, conducted over telephone or Zoom. These interviews will be used to collect data from Canadian EPI program team leads, Clinical Nurse Educators, clinical planners, clinicians, or managers, whose programs provide rural and remote EPI services within their community. Information collected will be further condensed into specific categories in order to identify the following: (1) commonalities and differences in the services provided, (2) program inclusion and exclusion criteria, (3) EPI team make up, (4) how services are accessed, (5) how services are delivered, (6) facilitators and barriers of care for rural EPI clients, and (7) recommendations for new EPI services being developed.

Participants can contact Ashley at Ashley.Forbes@vch.ca if they would like to withdraw their data.

V. Study Results

The results of this study will be reported in a graduate capstone report and main study findings may also be published in academic journal articles.

VI. Potential Benefits of the Study

We want to offer you the opportunity to be included in our prize draws (a $50 prepaid Visa card for Qualtrics survey participants and a $50 prepaid Visa card for semi-structured interview participants) by adding your contact email in question #1 of the EPI Qualtrics Survey. Your name and email will remain confidential.

VII. Confidentiality

Your confidentiality will be respected. Information that discloses your identity will not be released without your consent. Participant identities will be protected during and after this research study by use of numbered titles (i.e. Participant 01, Participant 02, etc.). These numbered titles will be used on all data collection forms. All documents will be kept secure in EPI Coastals' VCH password protected and encrypted drive made accessible only to this study's principal investigator and co-investigators listed on page 1.

Completion of the EPI Qualtrics Survey and/or selecting YES to question #22 of this survey indicates your consent to participate in this study. It will therefore be assumed that consent has been given.

VIII. Contact for Information about the Study

If you have any questions or concerns, please contact our team lead or practicum student. Their names and telephone numbers are listed on page 1 of this form.

IX. Contact for Complaints

If you have any concerns or complaints about your rights as a research participant and/or your experiences while participating in this study, contact the Research Participant Complaint Line in the UBC Office of Research Ethics at 604-822-8598 or if long distance e-mail RSIL@ors.ubc.ca or call toll free 1-877-822-8598.
